# Extracurricular activities as a moderator of the association between screen time and executive functions in children

**DOI:** 10.3389/fpsyg.2026.1843739

**Published:** 2026-08-12

**Authors:** Elena Chichinina, Anastasia Yakushina, Maria Dmitrieva, Natalia Rudnova, Aleksander Pashenko, Suncica Pocek, Patrik Drid

**Affiliations:** 1 Laboratory of Childhood Psychology and Digital Socialization, Federal Scientific Center of Psychological and Multidisciplinary Research (FSC PMR), Moscow, Russia; 2 Faculty of Psychology, Lomonosov Moscow State University, Moscow, Russia; 3 Faculty of Sport and Physical Education, University of Novi Sad, Novi Sad, Serbia; 4 Faculty of Medicine, University of Novi Sad, Novi Sad, Serbia

**Keywords:** executive functions, extracurricular activities, family income, maternal education, preschool age, screen time

## Abstract

**Introduction:**

Screen time and extracurricular activities represent two prominent components of modern childhood, particularly among children aged 6–7 years. Given the landscape, the aim of this study was to examine whether the number of different extracurricular activities attended by a child moderates the association between screen time and executive functions in children aged 6–7 years. Family income and maternal education were included as control variables, given their established links with children’s executive functions.

**Methods:**

The sample comprised 1,138 children aged 6–7 years (583 boys and 555 girls). Screen time, maternal education, family income, the number of different types of extracurricular activities a child attended, as well as characteristics of extracurricular activity participation were assessed using a questionnaire for mothers. Executive functions were assessed using subtests from the NEPSY-II neuropsychological battery and the Dimensional Change Card Sort. Analyses were conducted separately for boys and girls.

**Results:**

A significant moderating effect emerged exclusively for girls. The number of extracurricular activities moderated the relationship between screen time and verbal working memory, with low to moderate levels of activity participation strengthening the negative association. However, this finding should be interpreted with caution and is considered hypothesis-generating rather than confirmatory.

**Discussion:**

Among 6- to 7-year-old girls, the potential adverse effects of screen time on executive functions may be offset by a rich developmental environment, but firm conclusions cannot be drawn. Engagement in a diverse range of extracurricular activities may represent a plausible strategy for supporting verbal working memory in the context of elevated screen time, but these preliminary findings require independent replication before any practical recommendations can be made. For parents and educators, this study suggests that alongside limiting screen exposure, ensuring that children’s leisure time includes varied structured activities could be valuable—although this remains to be confirmed in future research.

## 1 Introduction

Executive functions (EFs) are a set of cognitive processes that enable voluntary control of behavior and thought, supporting adaptive behavior in novel situations (Diamond, 2012; Miyake et al., 2000). EFs comprise inhibition, working memory (visual and verbal), and cognitive flexibility (Miyake et al., 2000). During early childhood, EFs develop rapidly and are crucial for school readiness, as they underpin cognitive, social, and emotional development (Huang et al., 2020; Klopotova and Yaglovskaya, 2024; Sidneva, 2025). It is therefore important that children have well developed EFs by the end of the preschool period. In Russia, children typically enter school at approximately 7.5 years of age. In the period just before school entry (that is, at ages 6–7 years) children’s EFs development is shaped by multiple factors, including the kindergarten environment, peer interactions, various family characteristics, children’s screen time, and the extracurricular activities they attend (Diamond, 2012; Lakicevic et al., 2025). Of these factors, extracurricular activities have received little research attention, despite being a significant part of modern children’s lives (Gavrilova et al., 2025; RIA Novosti, 2022). This study focused on the association between screen time and EFs, and the previously unexplored moderating role of extracurricular activities in this association.

### 1.1 Screen time in children

According to World Health Organization recommendations, screen time for children aged 6–7 years should be limited to 2 h per day (Nuvoli et al., 2025). In practice, screen time among children of this age averages approximately 3 h per day, with higher usage on weekends than on weekdays (Liu et al., 2022; Muppalla et al., 2023). Screen time also varies by both duration and content depending on sex. Preschool aged boys tend to have higher average screen time than girls, largely due to time spent on digital games (Lakicevic et al., 2025). However, several studies have found no sex differences in screen time duration among children aged 5–7 years (Chakhunashvili et al., 2024; Veraksa et al., 2024). Empirical evidence indicates that screen time is positively correlated with family socioeconomic status (Pons et al., 2020; Veraksa et al., 2024). This pattern, however, is not universal across countries. In some contexts (China, for example) the opposite association has been observed (Hu et al., 2020; Ke et al., 2023). Thus, when examining screen time, it is important to consider variations by day of the week, account for sex differences, and control for family socioeconomic status.

Many researchers aim to differentiate the screen time. For instance, some distinguish between passive screen time (watching video content) and active screen time (interactive use of digital devices; Lakicevic et al., 2025). Others differentiate screen time by the type of digital device used (Moorman et al., 2019). Still others focus on screen time dedicated to specific purposes, such as education or entertainment (Blumberg et al., 2024; Uğraş et al., 2025). However, differentiating screen time is increasingly challenging due to the rapid development of the diverse functionalities offered by digital devices. For example, games and applications can range from simple, offline, single-user formats to online, multiplayer experiences with complex mechanics, incorporating artificial intelligence, extended reality, augmented reality, or virtual reality (Blumberg et al., 2024; Uğraş et al., 2025). Video viewing is also not a homogeneous activity: watching a feature film and scrolling through short-form videos (e.g., reels) represent fundamentally different forms of leisure. Moreover, children may use multiple digital devices simultaneously—for example, playing on a smartphone while a cartoon is playing on television in the background. Given this diversity of screen time modalities, we consider it appropriate to focus on total screen time duration unless the research aims specifically to examine particular types of digital activities.

It is important to note that screen time differs not only in content but also across sex. Although some studies have reported no significant sex differences in total screen time among children aged 5–7 years (Veraksa et al., 2024; Downing et al., 2017), other studies have identified stable sex-specific patterns of digital media use (Bustamante et al., 2023; Hu et al., 2020). In particular, longitudinal evidence suggests that school-aged boys spend more time using screens than girls, especially for video gaming (Hu et al., 2020). Similarly, Huang et al. (2025) demonstrated that the association between parenting style and problematic digital media use is mediated by EFs and differs between boys and girls.

A number of studies report that screen time is negatively associated with the development of EFs in older preschoolers (Bal et al., 2024; Lakicevic et al., 2025). A study by Lakicevic et al. (2025) on a sample of preschoolers aged 5–6 years found significant negative associations between screen time and cognitive flexibility, verbal working memory, and inhibition. Similarly, Hu et al. (2020) found that passive screen time negatively affects cognitive development in 5 year olds, specifically EFs, mathematical abilities, and vocabulary. Since EFs predict academic achievement and socio-emotional well-being (Emslander and Scherer, 2022; Huang et al., 2020; Sidneva, 2025), it is of particular interest to examine factors in children’s daily lives that may mitigate the negative effects of screen time on EFs. One way to minimize the negative impact of screen time on EFs may be children’s participation in extracurricular activities.

### 1.2 Extracurricular activities as a moderator of the association between screen time and EFs in children

The hypothesis that extracurricular activities may buffer the negative association between screen time and EFs is grounded in two complementary mechanisms. The first mechanism derives from the displacement hypothesis (Putnick et al., 2023). A substantial proportion of preschool children regularly participate in organized activities such as sports, music, and dance. In Russia, for example, parent surveys indicate that approximately 65% of children attend extracurricular activities (RIA Novosti, 2022). Consequently, extracurricular activities constitute a meaningful component of children’s daily routines and may shape developmental outcomes. Participation in such activities may reduce the amount of time available for screen use, thereby limiting exposure to potentially detrimental forms of digital media consumption.

The second mechanism is based on the cognitive enrichment hypothesis (Schoentgen et al., 2020). Following instructions, sustaining attention, and inhibiting impulsive responses—all common demands of extracurricular activities—engage the prefrontal cortex and anterior cingulate cortex, which are central neural substrates of EFs (Friedman and Robbins, 2022). Consistent with this perspective, previous studies have shown that children who regularly participate in extracurricular activities demonstrate higher levels of inhibition, working memory, and cognitive flexibility (Giordano et al., 2021; Rodriguez-Gomez and Talero-Gutiérrez, 2022). Thus, extracurricular activities may create a form of cognitive reserve that protects against risks associated with excessive screen exposure. Repeated cognitive engagement may strengthen neural circuits that are particularly vulnerable to the fragmented and rapidly shifting attentional demands associated with excessive screen use.

It should be emphasized that these two mechanisms do not function independently, but operate concurrently and synergistically, thereby warranting their integrated consideration in the analysis of the protective effects of extracurricular activities. A combined examination of these mechanisms offers a more complete account of how extracurricular activity can fulfill a buffering role against the adverse consequences of excessive screen time.

Moreover, different extracurricular activities impose distinct cognitive, social, and motor demands. Participation in only one type of activity may foster a relatively narrow set of skills, whereas engagement in diverse activities may support the development of EFs. For example, music and dance classes, which are more commonly attended by girls, place substantial demands on verbal working memory (Bayanova et al., 2023; Bergman Nutley et al., 2014; Shen et al., 2020). Sports activities, which are more popular among boys, may contribute more strongly to the development of inhibition and visuospatial abilities (Giordano et al., 2021). Chess, in turn, primarily trains planning and visuospatial working memory (Yakushina et al., 2025a). Therefore, participation in a broader range of extracurricular activities may facilitate a more balanced development of EFs.

Although several studies suggest that participation in a greater number of extracurricular activities is associated with better verbal working memory (Gavrilova et al., 2025), other findings support a curvilinear association. Specifically, a moderate level of participation appears beneficial, whereas beyond a certain threshold (typically three to four activity types) the advantages may plateau or even reverse because of fatigue, stress, and reduced opportunities for free play (Ren et al., 2020). In addition, extracurricular activities may mediate the association between parental education and children’s cognitive skills, particularly verbal working memory (Shatskaya et al., 2025). Despite accumulating evidence that extracurricular activities are beneficial for EFs, it remains unclear whether they also moderate the association between screen time and EFs development in preschoolers. Importantly, the specific cognitive demands of extracurricular activities may determine which EFs are protected from the negative effects of screen time. For example, activities that heavily engage verbal working memory (e.g., music and dance) may specifically buffer the association between screen time and verbal working memory, whereas activities that train inhibition or cognitive flexibility may buffer associations involving those EFs. This activity-specific hypothesis guided our decision to examine moderation effects separately for different EFs outcomes.

### 1.3 Current research

The aim of this study was to examine the number of different extracurricular activities attended by a child as a potential moderator of the association between screen time and EFs in children aged 6–7 years. The research question was formulated as follows: Does the association between screen time and EFs depend on the number of extracurricular activities a child attends?

We chose to focus on the number of different types of extracurricular activities for several reasons. First, today’s families have access to a wide variety of options for children, and it is common for a 6- to 7-year-old to participate in multiple types of extracurricular activities (RIA Novosti, 2022). Second, attending multiple types of extracurricular activities exposes the child to diverse experiences: both in terms of the activities themselves and in terms of interactions with instructors and peers. Third, participating in multiple types of extracurricular activities contributes to a structured weekly routine. We focused specifically on children aged 6–7 years because, in Russia, children at this age are in their final year of kindergarten. By the end of this period, it is crucial that they have developed sufficient EFs, as these are essential for school readiness (Ruffini et al., 2024).

Family income and maternal education were included as control variables, as these have been shown to be closely associated with children’s EFs (Hackman et al., 2015; Sarsour et al., 2011). Differences in the content and structure of activities, shaped by gendered parenting practices and biological influences (Berenbaum et al., 2007), may lead to differences in the development of EFs (Shinohara and Moriguchi, 2021), screen time (Lakicevic et al., 2025), and patterns of extracurricular activity participation between boys and girls. Moreover, boys and girls tend to choose different types of activities, so the same number of activities may have different developmental meanings for each sex. For this reason, we adopted a sex-stratified approach to our analysis.

## 2 Methods

### 2.1 Sample

Complete data on both EFs assessments and all maternal questionnaire items were required for inclusion. Participants with any missing values on these measures were excluded listwise, resulting in a final sample. Thus, the sample consisted of 1,138 children aged 6–7 years, including 583 boys (age: M = 79.9 months, SD = 5.15) and 555 girls (age: M = 79.9 months, SD = 4.75). All children attended the final year of kindergarten (preparatory groups) in municipal kindergartens across five Russian regions: 55.7% came from the city of Moscow; 18.4% from various localities in the Republic of Sakha (Yakutia); 14.6% from various localities in the Republic of Tatarstan; 5.9% from various localities in Perm Krai; and 5.4% from the city of Sochi.

All children participated in a standardized kindergarten curriculum for the final year of kindergarten, which includes the following 30-min classes: physical education (three times per week), music (twice per week), arts (once per week), crafts (once per week), math (twice per week), science (once per week), and writing and reading (twice per week; Veraksa et al., 2019).

### 2.2 Measures

#### 2.2.1 Measures: predictor variable. Weekly screen time

Screen time was assessed using a questionnaire for mothers. Mothers reported the average amount of time their child spent using digital devices separately on weekdays and on weekends, from which weekly screen time was calculated. We acknowledged that this measure captures only total duration, not content (e.g., passive vs. active, educational vs. entertainment) or device type (smartphone, tablet, television, computer). Using a maternal questionnaire is a convenient and widely used method for measuring screen time in young children, given that preschoolers have a limited understanding of time and typically do not yet possess their own personal devices. However, we recognize that questionnaire reports can be subject to biases (e.g., based on how busy the parent is or how much time they spend around their child).

#### 2.2.2 Measures: confound control variables. Maternal education and family income

Maternal education and family income were also assessed using the same questionnaire. Mothers reported their family’s income level by choosing one of four options: “below average,” “average,” “above average” or “other.” They also reported their own educational level, which was subsequently recoded into the number of years of education completed since first grade. The four-category item for family income provides only a crude estimate of socioeconomic status. It is worth noting that questions about income are widely recognized as highly sensitive in survey research, including in the Russian context. Respondents often refuse to answer such questions or provide biased estimates due to privacy concerns and social desirability effects (Andreenkova, 2017). The single four-category item used in this study was designed to mitigate these refusals by avoiding direct requests for exact monetary amounts.

#### 2.2.3 Measures: moderator variable. Number of extracurricular activities types

The number of different types of extracurricular activities types a child attended was also measured using the questionnaire. In the section of questions on extracurricular activities, mothers completed a table listing the following activity types: dance, music, sports, literacy and math, arts and crafts, foreign language, chess, robotics, acting, and other. For any extracurricular activity type the child did not attend, mothers indicated “does not attend.” For each extracurricular activity type the child did attend, mothers completed the corresponding row, specifying the specific activity (e.g., “soccer” for sports), the duration of attendance (in months), the length of a single session (in minutes), and the frequency of sessions (times per week).

Based on data collected via the parent questionnaire, we calculated the number of different types of extracurricular activities a child attended, as well as several characteristics of extracurricular activity participation: total weekly duration of all sessions (in minutes), the longest period of continuous attendance (in months), and total weekly frequency of all sessions (times per week).

We focused on the number of different activity types as the primary moderator because it captures diversity of cognitive demands. While we also collected data on total weekly duration, weekly frequency, and longest continuous attendance (reported in Table 1), we did not test these alternative characteristics as moderators. This decision is discussed in the Limitations section.

**Table 1:** Descriptive statistics of the study variables.

Study variables	Boys		Girls		Comparison of boys and girls			
	*Median*	*Min / Max*	*Median*	*Min / Max*	*U*	*r_b_*		*p*
Weekly screen time	980	30/5,580	840	20/4,680	155,990	0.10		0.003
Maternal education	14	9/20	14	9/20	172,496	0.01		0.876
Number of extracurricular activities types	2	0/7	2	0/7	159,732	0.01		0.704
Verbal working memory	19	5/34	19	7/32	166,810	0.04		0.259
Inhibition	11	1/19	12	2/19	156,380	0.10		0.003
Cognitive flexibility	20	9/24	21	10/24	164,052	0.05		0.108
The total duration of all session (in minutes per week)	225	20/1,090	260	10/1,460	100,773	0.09		0.013
The longest period of continuous attendance (in months)	24	0/60	24	0/62	107,964	0.07		0.068
The total frequency of all sessions (times per week)	4	0/17	4	0/24	108,109	0.07		0.075
	*%*		*%*		χ^2^	*df*	*V*	*p*
Family income								
below average	3.2		4.2		5.47	2	0.07	0.065
average	75.8		79.9					
above average	21.0		15.9					
Type of extracurricular activities								
Dance	10.8		34.4		91.6	1	0.28	< 0.001
Music	9.4		14.7		7.7	1	0.08	0.006
Sports	62.6		38.2		68.7	1	0.25	< 0.001
Literacy and math	57.5		54.4		1.1	1	0.03	0.290
Arts and crafts	10.1		19.7		21.1	1	0.14	< 0.001
Foreign language	15.7		15.3		0.1	1	0.01	0.837
Chess	17.5		7.2		28.1	1	0.16	< 0.001
Robotics	7.5		0.9		30.4	1	0.16	< 0.001
Acting	2.6		5.6		6.7	1	0.08	0.010
Other	4.4		4.7		0.1	1	0.01	0.851

#### 2.2.4 Measures: outcome variables. EFs

EFs were assessed using subtests from the NEPSY-II neuropsychological battery (Korkman et al., 2007) and the Dimensional Change Card Sort (Zelazo, 2006). These tests have been validated on Russian samples (Veraksa et al., 2020).

Inhibition was assessed using the Inhibition subtest (score range: 0–20). In this task, children are presented with a series of 40 geometric shapes (squares and circles). In the first condition, children name the shapes as quickly as possible. In the second condition, children perform an inhibition task, naming the shapes in reverse (e.g., saying “circle” for a square and vice versa) as quickly as possible. A practice trial is administered before each condition. Completion time, the number of uncorrected errors, and the number of corrected errors are recorded, and a composite score is calculated based on these parameters.

Verbal working memory was assessed using the Sentence Repetition subtest (score range: 0–34). In this task, children are asked to repeat sentences that increase in length and syntactic complexity. There are 17 sentences in total. Each correctly repeated sentence receives 2 points; a sentence repeated with one or two errors receives 1 point; and a sentence repeated with more than two errors receives 0 points. If a child receives 0 points on four consecutive sentences, the task is discontinued.

Cognitive flexibility was assessed using the Dimensional Change Card Sort (score range: 0–24). In this task, children are presented with cards depicting boats and rabbits on a white background; both boats and rabbits can be either blue or red. Some cards have a black border. First, children sort six cards by color (placing blue cards on one side and red cards on the other). Next, they sort six cards by content (placing cards with rabbits on one side and cards with boats on the other). Finally, they sort cards by the presence or absence of a black border, regardless of the card’s color or content. One point is awarded for each correctly sorted card.

### 2.3 Procedure

EFs assessment was conducted during a 20-min session in the morning, in a quiet, well-lit room at the child’s kindergarten. Children were free to decline participation at any point during the assessment if they did not wish to continue.

During the same month as the assessments, an online questionnaire was administered to the mothers of the participating children. A link to the questionnaire was distributed via email and through parent messaging groups associated with the kindergartens. Completing the questionnaire took approximately 10 min.

Prior to the study, written informed consent was obtained from parents or legal guardians for their child’s participation. This study was conducted in accordance with the ethical standards laid down in the Declaration of Helsinki. The study was reviewed and approved by the Ethics Committee of the Federal Scientific Center for Psychological and Interdisciplinary Research (Approval No. 3, January 10, 2025).

### 2.4 Data analysis

Statistical analyses were performed using Jamovi version 2.0.0.0. The normality of the distribution was examined with the Shapiro–Wilk test. Since the data were not normally distributed, non-parametric tests were employed.

First, descriptive statistics (median, minimum, maximum) were calculated for the predictor, control, moderator, and outcome variables, as well as for variables describing the characteristics of extracurricular activity participation. This allowed us to examine not only the number of extracurricular activities but also different characteristics of extracurricular activities participation (the total length of all session in minutes per week, the longest period of continuous attendance in months, the total weekly frequency of all sessions in times per week). For all continuous variables, boys and girls were compared using the Mann–Whitney test (*U*). Rank biserial correlation (*r_b_*) was used to calculate effect sizes for Mann–Whitney test with the following thresholds: 0.09 < *r_b_* < 0.30 small, 0.29 < *r_b_* < 0.50 moderate, *r_b_* ≥ 0.50 large (Cohen, 2013). Chi-square test (χ2) as used to compare boys and girls on nominal and ordinal variables. Cramér’s *V* was used to calculate effect sizes for the chi square test. For tests with one degree of freedom, thresholds were: 0.09 < *V* < 0.30 small, 0.29 < *V* < 0.50 moderate, and *V* ≥ 0.50 large. For tests with two degrees of freedom, thresholds were: 0.06 < *V* < 0.21 small, 0.20 < *V* < 0.35 moderate, and *V* ≥ 0.35 large (Cohen, 2013).

Second, to examine the data structure and rule out multicollinearity prior to moderation analysis, Spearman’s correlations were calculated between the predictor, control, moderator, and outcome variables. Thresholds for interpreting Spearman’s correlation coefficients were as follows: 0.09 < *ρ* < 0.40 weak, 0.39 < *ρ* < 0.71 moderate, and *ρ* ≥ 0.70 strong (Dancey and Reidy, 2007).

Third, moderation analysis was conducted to test the hypothesized model in which the association between screen time and EFs depended on the number of extracurricular activities. Because this study was exploratory and involved multiple statistical tests (3 EFs outcomes × 2 sex-stratified groups × 5 predictors / interactions = 30 tests), we did not apply a formal correction for multiple comparisons (e.g., FDR or Bonferroni). Such corrections would be overly conservative for discovery-oriented research and could mask potentially interesting but small effects. Instead, we report all *p*-values and effect sizes exactly as obtained and interpret significant findings with caution, emphasizing the need for replication.

## 3 Results

### 3.1 Descriptive statistics for all study variables

Table 1 reports descriptive statistics for the predictor, control, moderator, and outcome variables, as well as for the characteristics of extracurricular activities participation. Screen time was lower for girls than for boys, with boys averaging 980 min per week (approximately 16 h) and girls averaging 840 min per week (14 h; Table 1). Girls attended a statistically significant greater number of extracurricular activity types than boys (Table 1). Girls demonstrated significantly higher levels of verbal working memory and inhibition than boys (Table 1).

With respect to the characteristics of extracurricular activities participation, the following sex differences emerged (Table 1). Girls had a higher total weekly duration of extracurricular activities than boys: 4 h and 20 min per week compared to 3 h and 45 min, respectively. Girls were significantly more likely to attend dance, music, art, and acting classes, whereas boys were significantly more likely to attend sports, chess, and robotics.

Table 2 reports the distribution of the number of extracurricular activity types attended in boys and girls.

**Table 2:** Number of extracurricular activities types in boys and girls.

Number of extracurricular activities types	Boys	Girls	Comparison of boys and girls			
	*%*	*%*	χ^2^	*df*	*V*	*p*
No extracurricular activities	13.0	16.8	8.44	8	0.09	0.391
One type	26.8	24.5				
Two types	29.9	26.5				
Three types	17.2	18.2				
Four types	8.2	9.3				
Five types	4.1	3.1				
Six types	0.5	0.7				
Seven types	0.3	0.5				

### 3.2 Correlation results

Table 3 reports Spearman’s correlations between the predictor, moderator, outcome, and control variables, shown separately for boys and girls. Correlational analysis revealed no strong associations between the number of extracurricular activities types and screen time, indicating that multicollinearity was not a concern. The absence of multicollinearity provided the methodological basis for testing a moderation model with screen time as the predictor and number of extracurricular activities as the moderator.

**Table 3:** Correlation matrix among the study variables.

Study variables	Sex	1.	2.	3.	4.	5.	6.
1. Weekly screen time	Boys						
	Girls						
2. Maternal education	Boys	−0.223***					
	Girls	−0.204***					
3. Family income	Boys	−0.075	0.103*				
	Girls	−0.114**	0.067				
4. Number of extracurricular activities types	Boys	−0.301***	0.219***	0.137***			
	Girls	−0.360***	0.158***	0.112**			
5. Verbal working memory	Boys	−0.258***	0.139***	0.062	0.231***		
	Girls	−0.233***	0.129**	0.038	0.242***		
6. Inhibition	Boys	−0.069	−0.030	−0.005	0.044	0.227***	
	Girls	−0.014	−0.064	0.077	0.082	0.202***	
7. Cognitive flexibility	Boys	−0.120**	0.019	0.039	0.099*	0.415***	0.235***
	Girls	−0.135**	0.075	0.045	0.098*	0.431***	0.258***

1 – Weekly screen time, 2 – Maternal education, 3 – Family income, 4 – Number of extracurricular activities types, 5 – Verbal working memory, 6 – Inhibition, 7 – Cognitive flexibility; * *p* ≤ 0.05, ** *p* ≤ 0.01, *** *p* ≤ 0.001.

### 3.3 Moderation results

Table 4 reports the relationships between screen time, control variables (maternal education and family income), number of extracurricular activities types, and EFs. The control variables almost did not predict EFs: only in boys, maternal education was a positive predictor of verbal working memory. In boys and girls, screen time negatively predicted verbal working memory, also in girls screen time negatively predicted cognitive flexibility.

**Table 4:** Moderation effect of number of extracurricular activities types on the relationships between screen time and EFs in boys and girls.

Model variables	Boys					Girls				
	*B (SE)*	*t*	*p*	η^2^*p*	*95% CI*	*B (SE)*	*t*	*p*	η^2^*p*	*95% CI*
Verbal working memory as an outcome variable										
Intercept	18.923 (0.194)	97.34	< 0.001		18.541; 19.305	19.298 (0.184)	104.65	< 0.001		18.935; 19.660
Weekly screen time	- 0.001 (< 0.001)	−3.67	< 0.001	0.023	−0.002; −0.001	−0.001 (0.001)	−4.35	< 0.001	0.034	−0.002; −0.001
Maternal education	0.306 (0.122)	2.50	0.013	0.011	0.065; 0.546	0.216 (0.127)	1.70	0.090	0.005	−0.034; 0.467
Family income	0.171 (0.412)	0.41	0.679	< 0.001	−0.639; 0.980	−0.189 (0.406)	−0.47	0.642	< 0.001	−0.986; 0.609
Number of extracurricular activities types	0.528 (0.146)	3.62	< 0.001	0.022	0.242; 0.814	0.525 (0.131)	4.01	< 0.001	0.029	0.268; 0.783
Weekly screen time * Number of extracurricular activities types	- 0.001 (0.001)	−0.23	0.819	< 0.001	−0.001; < 0.001	0.001 (0.001)	2.85	0.005	0.015	< 0.001; < 0.001
Model info	*F* (5, 571) = 11.47, *p* = 0.089, adj. *R*^2^ = 0.08					*F* (5, 545) = 15.45, *p* < 0.001, adj. *R*^2^ = 0.12				
Inhibition as an outcome variable										
Intercept	11.049 (0.143)	77.08	< 0.001		10.768; 11.331	11.539 (0.140)	82.38	< 0.001		11.264; 11.814
Weekly screen time	−0.001 (< 0.001)	−1.08	0.280	0.002	−0.001; < 0.001	- 0.001 (< 0.001)	−1.51	0.133	0.004	−0.001; < 0.001
Maternal education	−0.087 (0.090)	−0.97	0.333	0.002	−0.264; 0.090	−0.144 (0.097)	−1.48	0.139	0.004	−0.334; 0.047
Family income	−0.082 (0.304)	−0.27	0.788	< 0.001	−0.679; 0.515	0.568 (0.308)	1.84	0.066	0.006	−0.038; 1.174
Number of extracurricular activities types	0.069 (0.107)	0.65	0.518	0.001	−0.141; 0.280	0.123 (0.100)	1.23	0.219	0.003	−0.073; 0.318
Weekly screen time * Number of extracurricular activities types	< 0.001 (< 0.001)	0.27	0.790	< 0.001	−0.001; < 0.001	−0.001 (< 0.001)	−1.11	0.267	0.002	−0.001; < 0.001
Model info	*F* (5, 571) = 0.59, *p* = 0.707, adj. *R*^2^ = 0.01					*F* (5, 545) = 2.26, *p* = 0.047, adj. *R*^2^ = 0.01				
Cognitive flexibility as an outcome variable										
Intercept	20.024 (0.125)	159.81	< 0.001		19.777; 20.270	20.366 (0.117)	174.08	< 0.001		20.136; 20.596
Weekly screen time	−0.001 (< 0.001)	−1.24	0.216	0.003	−0.001; < 0.001	−0.001 (< 0.001)	−2.02	0.044	0.007	−0.001; −0.001
Maternal education	0.063 (0.079)	0.80	0.427	0.001	−0.092; 0.217	0.098 (0.081)	1.21	0.228	0.003	−0.061; 0.257
Family income	0.064 (0.266)	0.24	0.810	< 0.001	−0.458; 0.586	0.211 (0.257)	0.82	0.413	0.001	−0.295; 0.717
Number of extracurricular activities types	0.124 (0.094)	1.33	0.186	0.003	−0.060; 0.309	0.053 (0.083)	0.64	0.526	0.001	−0.111; 0.216
Weekly screen time * Number of extracurricular activities types	< 0.001 (< 0.001)	0.51	0.609	< 0.001	−0.001; < 0.001	< 0.001 (< 0.001)	1.83	0.068	0.006	−0.001; < 0.001
Model info	*F* (5, 571) = 1.59, *p* = 0.162, adj. *R*^2^ = 0.01					*F* (5, 545) = 3.34, *p* = 0.006, adj. *R*^2^ = 0.02				

**Figure 1:**
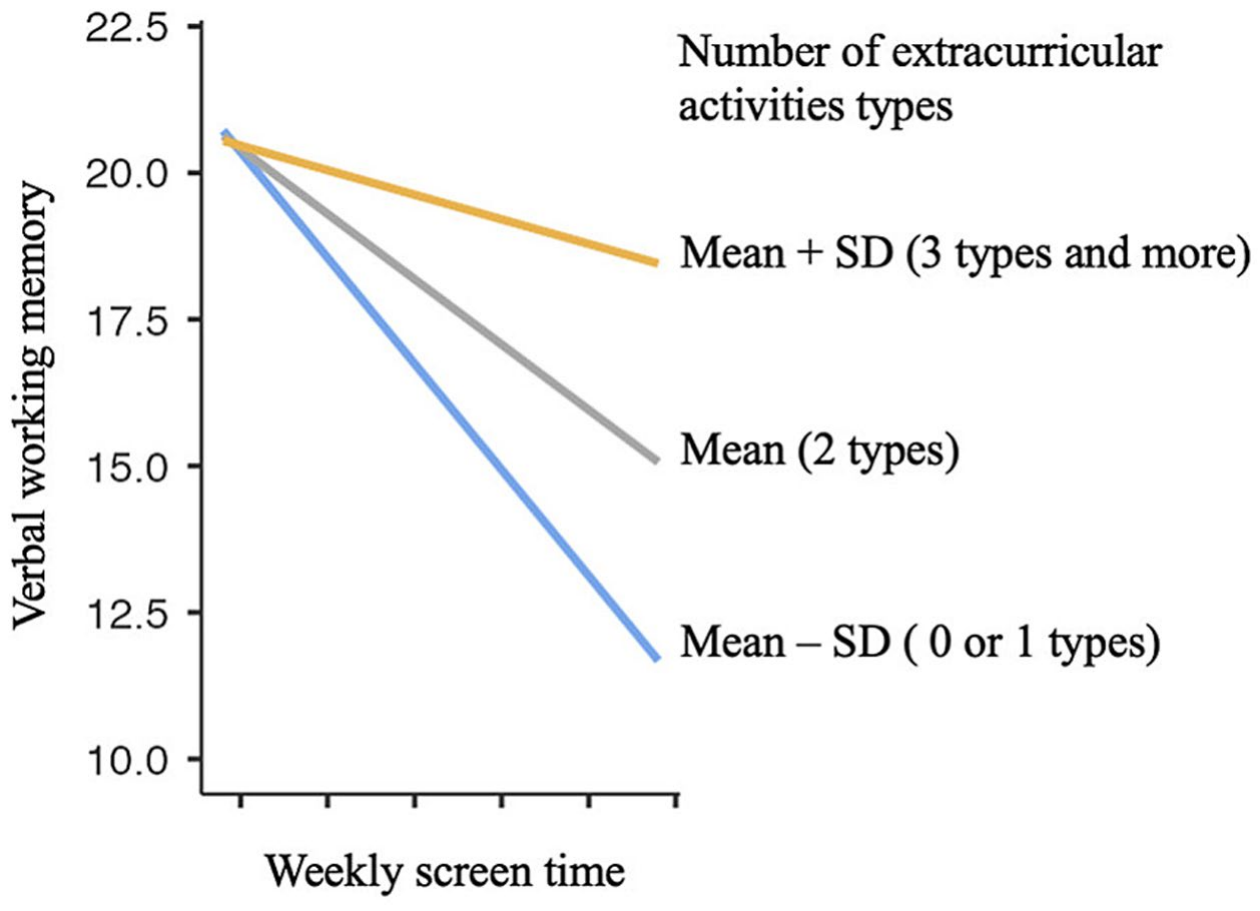
Conditional effects of screen time on verbal working memory by extracurricular activity levels in girls.

There was one significant interaction, only for girls (Table 4). Number of extracurricular activities moderated the relationship between screen time and verbal working memory. Among girls, the mean number of activity types was M = 1.94 (SD = 1.42). Thus, a low number of activities (M − SD) ranged from 0 to 1 type, an average number (M) corresponded to 2 types, and a high number (M + SD) corresponded to 3 or more types. Low and average levels of extracurricular activity variety amplified the negative relationship between screen time and verbal working memory, whereas a high level did not statistically significantly moderate the screen time—EFs association. These decompositions are presented in Table 5 and Figure 1 for girls.

**Table 5:** Conditional effects of screen time on verbal working memory by extracurricular activity levels in girls.

Moderator variable level	*B (SE)*	*t*	*p*	η^2^*p*	*95% CI*
Low number of extracurricular activities types (Mean − SD)	−0.002 (< 0.001)	−5.81	< 0.001	0.058	−0.002; −0.001
Average number of extracurricular activities types (Mean)	−0.001 (< 0.001)	−4.35	< 0.001	0.034	−0.002; −0.001
High number of extracurricular activities types (Mean + SD)	−0.001 (< 0.001)	−1.07	0.284	0.002	−0.001; < 0.001

“Mean – SD” = 0 or 1 types of extracurricular activities, “Mean” = 2 types of extracurricular activities, “Mean + SD” = 3 and more types of extracurricular activities.

## 4 Discussion

Screen time among today’s 6- to 7-year-old children often exceeds recommended limits, thereby posing a threat to the development of EFs, which are crucial for school readiness, successful socialization, and academic learning (Emslander and Scherer, 2022; Ruffini et al., 2024). At the same time, children of this age frequently participate in extracurricular activities beyond the standard kindergarten curriculum, which may buffer the negative effects of screen time on EFs. However, the moderating role of extracurricular activities in the association between screen time and EFs has not yet been investigated. Accordingly, the aim of this study was to examine the number of different types of extracurricular activities a child attends as a potential moderator of the association between screen time and EFs in children aged 6–7 years. The research question was: Does the association between screen time and EFs depend on the number of extracurricular activities a child attends?

Our results indicate that among girls who participate in relatively few types of extracurricular activities (two or less), higher screen time is associated with lower verbal working memory scores. By contrast, among girls who participate in a broader range of extracurricular activities (three or more types), this negative association becomes substantially weaker and statistically non-significant. It is important to note, however, that although we identified significant moderation interaction for girls (*p* = 0.005, η^2^p = 0.015), the total number of statistical tests (*n* = 30) increases the risk of a Type I error. A strict correction for multiple comparisons (e.g., FDR) would set the critical *p*-value for the smallest test at 0.0017, and our result does not reach this threshold. Consequently, this finding should not be considered confirmatory. Independent replication is required before any practical recommendations can be made.

The observed moderation effect, which emerged only for low and average levels of extracurricular activity participation (but not for high levels), tentatively suggests that even a modest range of activities may serve as a developmental buffer, attenuating the negative association between screen time and verbal working memory in girls. Importantly, the results are consistent with the cognitive enrichment hypothesis, according to which cognitively stimulating environments contribute to the strengthening of EFs through repeated engagement of attention, memory, and self-regulation processes. At the same time, the observed moderation effect may also be interpreted through the lens of the displacement hypothesis. Children who regularly attend multiple extracurricular activities likely spend less unstructured time at home and therefore may have fewer opportunities for prolonged passive screen use. In this sense, extracurricular activities may influence EFs not only through cognitive stimulation itself, but also indirectly through the organization of children’s daily routines and leisure environments (Shatskaya et al., 2025).

An important conceptual implication of the present findings is that extracurricular activities do not appear to operate as a universally protective factor across all EFs. Instead, the buffering effect was specific to verbal working memory in girls. This pattern is consistent with the idea of domain-specific compensation: certain structured activities may preferentially strengthen those executive processes that are most actively engaged during participation. Such an interpretation aligns with previous evidence showing that music and dance activities place particularly strong demands on sequential auditory processing, retention of verbal instructions, timing, and coordinated behavioral control (Bayanova et al., 2023; Liu et al., 2025). Nevertheless, these insights should be viewed as hypothesis-generating rather than definitive, and they merit further investigation.

We suggest that our finding is related to the cognitive demands of the extracurricular activities that girls attend. As shown in the descriptive statistics, girls in our sample were likely to attend literacy and math classes (54%), sports (38%), dance (34%), arts and crafts (20%), foreign language (15%), and music (15%) classes. All of these activities except arts and crafts place a strong emphasis on auditory or verbal memory and accurate reproduction of the required content. Specifically, in literacy and math classes, children actively listen to the teacher and process most information aurally, as they are not yet able to read fluently. Among sports activities, in Russia, girls aged 6–7 years, most often attend gymnastics and rhythmic gymnastics or sport dances (VCIOM, 2023)—that is, music-based sports activities. In gymnastics and dance classes, one of the key skills is remembering and subsequently reproducing sequences of movements, often to a count or musical accompaniment. In music classes, children must hold in memory the melodic line, rhythmic pattern, and sequence of notes or lyrics while following the teacher’s voice or musical accompaniment (Bayanova et al., 2023; Bergman Nutley et al., 2014). This requires holding in working memory both the instruction itself and the execution of movements in time with the music (Shen et al., 2020). In foreign language classes, 6- to 7-year-old children primarily practice speaking and memorizing new words by ear, which also engages verbal working memory. Thus, the activities that girls attend place high demands on verbal working memory. Accordingly, we propose that the high demands placed on verbal working memory during extracurricular activities compensate for the negative effects of screen time on verbal working memory among girls (Gavrilova et al., 2025; Lakicevic et al., 2025).

This finding was not observed among boys, likely due to the activity types most common among boys in our sample: sports (63%), literacy and math (58%), chess (18%), and foreign language (16%). Although sports benefit EFs generally (Giordano et al., 2021), their impact on verbal working memory in boys may be weaker than in girls. Russian boys aged 6–7 typically attend soccer and martial arts (VCIOM, 2023)—activities that engage visuospatial rather than verbal working memory (Yakushina et al., 2025b). Chess also primarily develops visuospatial skills and planning (Gavrilova et al., 2025; Yakushina et al., 2025a). However, visuospatial working memory was not measured in this study.

In both boys and girls, the association between screen time and inhibition and cognitive flexibility was not moderated by the number of extracurricular activities types. Moreover, weekly screen time was not a predictor of inhibition, and was a negative predictor of cognitive flexibility in girls only. However, many studies have reported negative associations between screen time and inhibition, and between screen time and cognitive flexibility, in preschoolers (Hu et al., 2020; Lakicevic et al., 2025). One possible explanation for the absence of such associations in our study is that our models accounted for the number of extracurricular activities, maternal education, and family income. Nevertheless, even in our study, a weak negative correlation was observed between screen time and cognitive flexibility. The absence of a correlation between screen time and inhibition may be due to the fact that screen time in our sample was not excessively high: approximately 16 h per week for boys and 14 h for girls. Furthermore, the nature of children’s screen time in our study remains unknown; it is possible that by age 6–7, screen time largely consists of activities that do not harm inhibition, such as educational apps that children use together with their parents. Additionally, our results may differ from those reported in previous studies due to differences in the age of preschool participants. Specifically, the study by Hu et al. (2020) involved children aged 5 years, whereas our sample consisted of children aged 6–7 years. Given that a 1.5-year age gap during the preschool period can reflect substantial developmental changes in EFs, this discrepancy in age composition may account for at least part of the observed differences between the findings.

Family income and maternal education were almost unrelated to EFs, with only maternal education positively predicting boys’ verbal working memory. This is surprising given the extensive literature (Hackman et al., 2015; Sarsour et al., 2011) demonstrating robust associations between family socioeconomic status and children’s EFs. We attribute this to the bias in subjective socioeconomic reporting in sociological research: different individuals have very different perceptions of what constitutes “average” income. In our study, family income was measured based on mothers’ subjective selection of one of three response options—below average, average, or above average. This subjective measurement likely introduced systematic bias, as mothers with identical objective incomes may have categorized themselves differently depending on their reference groups and social comparisons. Therefore, the null findings regarding family characteristics should be interpreted with caution and do not necessarily contradict previous literature that used more objective income measures.

Taken together, the main finding of the study offers preliminary evidence that that extracurricular activities may operate not simply as a substitute for screen-based leisure, but as a context that provides cognitively and socially enriched experiences. Structured activities typically require sustained attention, rule-following, behavioral self-regulation, and interaction with instructors and peers. Such experiences may partially counterbalance the fragmented attentional patterns often associated with prolonged digital media exposure, although this interpretation remains preliminary. At the same time, the absence of significant moderation effects for inhibition and cognitive flexibility indicates that extracurricular activities are unlikely to function as a universal protective factor for EFs. Rather, we could suggest, that any protective effect depends on the correspondence between the cognitive demands of the extracurricular activity and the specific executive process being assessed.

## 5 Strength and limitations

Several limitations of this study should be acknowledged. The relatively large number of limitations reflects the exploratory nature of the study, and the complexity of measuring real-world behaviors (screen time and extracurricular activities) in a young child sample.

First, the cross-sectional design precludes any causal inferences. Reverse causality may operate: children with poorer verbal working memory might be given more screen time and enrolled in fewer extracurricular activities due to behavioral difficulties. Unmeasured variables such as parenting style, child temperament, or household chaos could also influence all three constructs. Longitudinal studies are needed to establish temporal precedence. Second, due to the exploratory design (30 tests, no pre-registered hypotheses), the sole significant interaction for girls does not withstand FDR correction and thus requires replication before any practical conclusions can be drawn. Third, the moderator was limited to the number of different extracurricular activities types, with no parallel tests of total duration, frequency, or intensity. While we provided descriptive statistics for these other characteristics, we did not examine whether they produce the same moderating effect. Future studies should compare different operationalizations of extracurricular activities participation. Also, future studies should explore potential non-linear patterns using GLM or machine learning approaches. Fourth, although we interpreted the sex-specific moderation effect as reflecting differences in the cognitive demands of typical extracurricular activities (dance/music for girls, sports/chess for boys), we did not directly assess the cognitive load of each extracurricular activity type. Our interpretation is plausible given prior research, but alternative explanations (e.g., differences in instructional quality or peer interactions) cannot be ruled out. Finally, generalizability may be limited by the specific cultural and educational context of Russia, where school entry occurs at approximately 7.5 years (later than in many Western countries) and extracurricular activities are often low-cost and municipally provided. Replication in other countries is needed.

Several strengths of the present study should be noted. First, we conducted sex stratified analyses, which recognizes that boys and girls may follow distinct developmental trajectories shaped by both biological and environmental factors (Berenbaum et al., 2007). Second, we addressed a topic that has received limited attention in the existing literature: the moderating role of extracurricular activities. This is particularly relevant given that such activities are highly prevalent among children aged 6 to 7 years. Third, the study benefited from a large sample across sex and geography, which enhances the generalizability of our findings.

## 6 Conclusion

The present study makes a contribution to understanding child development in the digital age. Its main finding is that among 6- to 7-year old girls, the negative effects of screen time on EFs may be mitigated by enriching the developmental environment. For parents and educators, our findings highlight the importance not only of limiting screen time, but also of filling children’s leisure with diverse structured activities. For girls, attending multiple extracurricular activities may support verbal working memory in the context of high screen time, though this conclusion requires replication. Future studies should consider the characteristics of screen time (e.g., passive vs. active, solitary vs. social, weekday vs. weekend) as well as structured family activities (e.g., shared reading, music making, games, visits to theaters and museums), rather than focusing solely on extracurricular activities.

## Data Availability

The raw data supporting the conclusions of this article will be made available by the authors, without undue reservation.
